# Reduced decay-accelerating factor expression promotes complement-mediated cystogenesis in murine ADPKD

**DOI:** 10.1172/jci.insight.175220

**Published:** 2024-05-23

**Authors:** Sofia Bin, Miran Yoo, Paolo Molinari, Micaela Gentile, Kelly Budge, Chiara Cantarelli, Yaseen Khan, Gaetano La Manna, William M. Baldwin, Nina Dvorina, Paolo Cravedi, G. Luca Gusella

**Affiliations:** 1 Translational Transplant Research Center and Department of Medicine, Icahn School of Medicine at Mount Sinai, New York, New York, USA.; 2 Nephrology, Dialysis and Kidney Transplant Unit, IRCCS Azienda Ospedaliero- University of Bologna, Italy.; 3 Department of Medical and Surgical Sciences (DIMEC), Alma Mater Studiorum - University of Bologna, Bologna, Italy.; 4 Division of Nephrology, Icahn School of Medicine at Mount Sinai, New York, New York, USA.; 5 Unità Operativa Nefrologia, Azienda-Ospedaliero University of Parma, Department of Medicine and Syrgery, University of Parma, Italy.; 6 Department of Inflammation and Immunity, Lerner Research Institute, Cleveland Clinic, Cleveland, Ohio, USA.

**Keywords:** Nephrology, Complement

## Abstract

Patients with autosomal dominant polycystic kidney disease (ADPKD), a genetic disease due to mutations of the *PKD1* or *PKD2* gene, show signs of complement activation in the urine and cystic fluid, but their pathogenic role in cystogenesis is unclear. We tested the causal relationship between complement activation and cyst growth using a Pkd1^KO^ renal tubular cell line and newly generated conditional *Pkd1^–/–^* *C3^–/–^* mice. *Pkd1*-deficient tubular cells have increased expression of complement-related genes (*C3*, *C5*, *CfB*, *C3ar*, and *C5ar1*), while the gene and protein expression of complement regulators DAF, CD59, and Crry is decreased. *Pkd1^–/–^* *C3^–/–^* mice are unable to fully activate the complement cascade and are characterized by a significantly slower kidney cystogenesis, preserved renal function, and reduced intrarenal inflammation compared with *Pkd1^–/–^* *C3^+/+^* controls. Transgenic expression of the cytoplasmic C-terminal tail of *Pkd1* in Pkd1^KO^ cells lowered *C5ar1* expression, restored *Daf* levels, and reduced cell proliferation. Consistently, both DAF overexpression and pharmacological inhibition of C5aR1 (but not C3aR) reduced Pkd1^KO^ cell proliferation. In conclusion, the loss of *Pkd1* promotes unleashed activation of locally produced complement by downregulating DAF expression in renal tubular cells. Increased C5a formation and C5aR1 activation in tubular cells promotes cyst growth, offering a new therapeutic target.

## Abstract 2

Complement system activation is implicated in the pathogenesis of the most common renal genetic disorder, autosomal dominant polycystic kidney disease.

## Graphical abstract

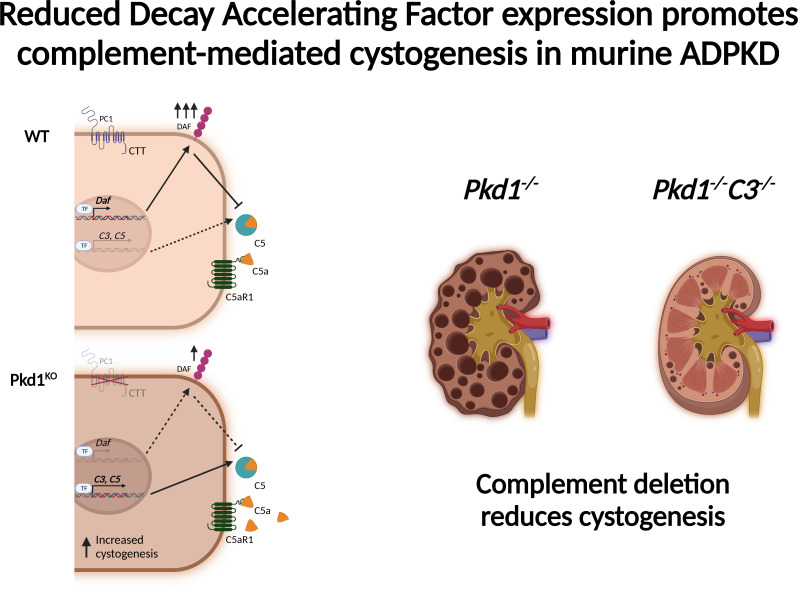

## Introduction

Autosomal dominant polycystic kidney disease (ADPKD) is the most common renal genetic disorder caused mainly by mutations of the *PKD1* or *PKD2* gene, which code for polycystin-1 (PC1) or polycystin-2 (PC2), respectively (1, 2). The disease is characterized by the development and growth of tubular cysts that undermine the renal architecture, leading to renal failure in 50% of patients by the age of 60 (3). ADPKD kidneys are in a state of chronic injury and inflammation due to progressive cyst expansion and tissue remodeling (4).

Complement components have been described in the urine and cystic fluid of ADPKD patients, suggesting that complement system activation may play a role in ADPKD pathogenesis (5–8). The complement system comprises over 30 circulating and membrane-bound proteins, activated as a proteolytic cascade through 3 different pathways: classical, lectin, and alternative. Complement split products act as opsonins, chemoattractants, and serve as modulators of innate immune cell function (8). More recently, complement has been shown to also modulate adaptive immune cell responses and nonimmune processes such as cell survival, proliferation, and metabolism (8).

Complement activation is tightly regulated to prevent bystander damage to self-cells (9). This regulation is accomplished through the expression of membrane-bound and soluble complement-regulating proteins. Among these, decay-accelerating factor (DAF or CD55) is a glycophosphatidylinositol-anchored, membrane-bound complement regulator that accelerates the decay of cell surface–assembled C3 convertases, thus limiting the downstream complement activation and formation of cleavage products, including C3a and C5a (10). Notably, DAF functions intrinsically, restricting complement activation only on the surface of the cells on which it is expressed. Data by our group and others have shown a critical role for DAF in limiting complement activation in numerous immunological processes and in glomerular diseases (11–13).

Other complement regulators include the surface-expressed membrane cofactor protein (MCP or CD46 in humans and Crry in mice) that has both decay-accelerating function and cofactor activity (8), CD59 that inhibits formation of the membrane attack complex (MAC or C5b-9), CR1, and the soluble complement factor H (CfH) (8).

Whether and how *PKD1* dysregulation affects complement activation, and how the complement system regulates cystogenesis remain unclear. Herein, we assessed the relationship between complement activation and cyst formation using both in vitro and in vivo *Pkd1*-deficient murine systems.

## Results

### Complement and complement regulators in Pkd1^–/–^ kidneys.

**Figure 1:**
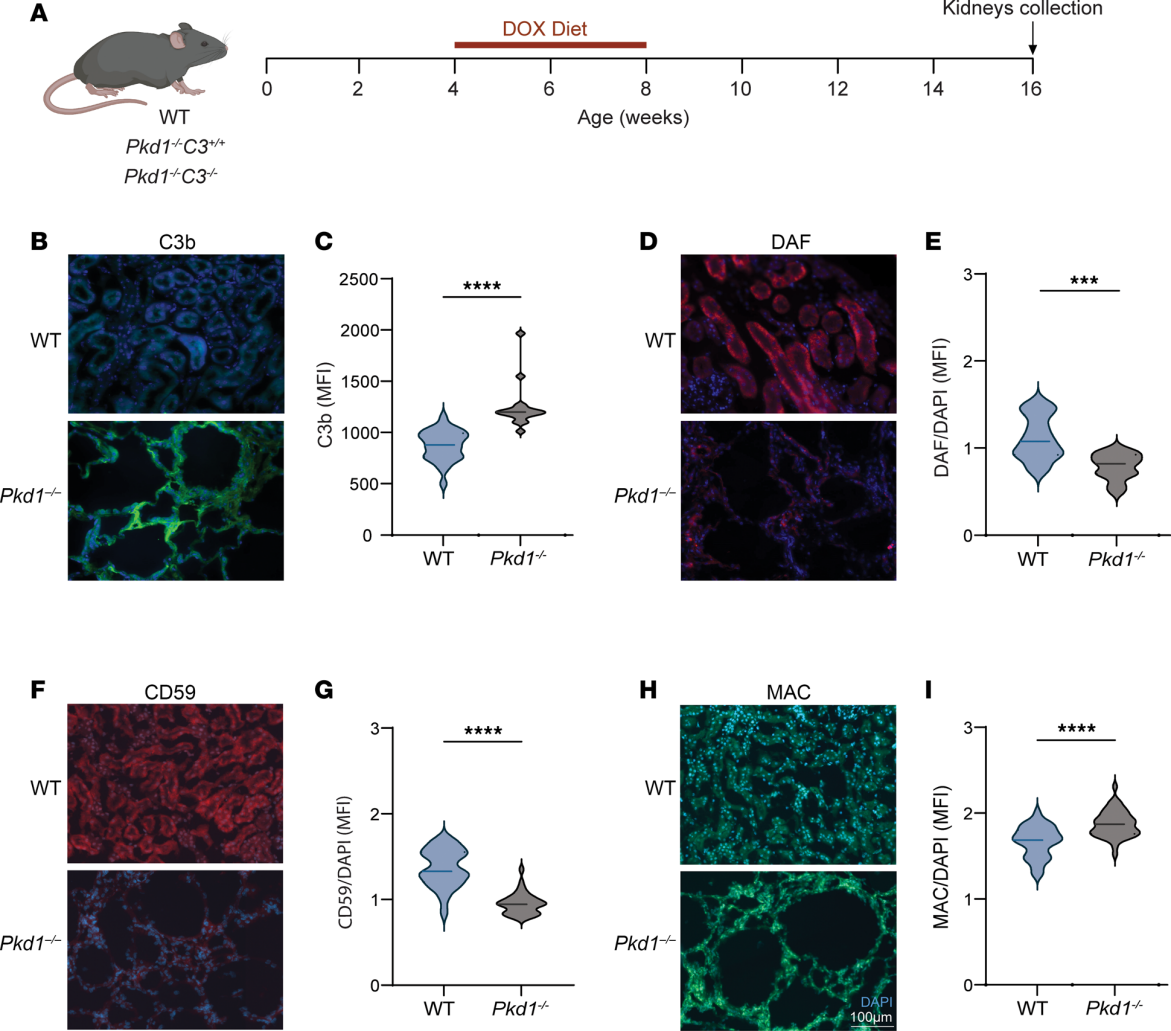
*Pkd1^–/–^* kidneys show signs of complement activation. (**A**) WT (*n* = 10), *Pkd1^–/–^* *C3^+/+^* (*n* = 9), and *Pkd1^–/–^* *C3^–/–^* (*n* = 14) animals were fed doxycycline-supplemented (DOX-supplemented) chow for 4 weeks, starting at 4 weeks of age. Kidneys were collected at weeks 16 of age. Immunofluorescence detection and corresponding quantification (MFI) of (**B** and **C**) C3b, (**D** and **E**) DAF, (**F** and **G**) CD59, and (**H** and **I**) MAC. ***P < 0.001, ****P < 0.0001 by unpaired, 2-tailed *t* test. Scale bar: 100 μm (applies to all images).

To test the role of complement in PKD progression, we used a slowly progressing conditional model of renal cystogenesis in which the *Pkd1* gene can be ablated by the expression of Cre recombinase under the control of the doxycycline-inducible (Dox-inducible) promoter (*Pax8^rtTA^;TetO-Cre;Pkd1^fl/fl^* mice; hereafter referred to as *Pkd1^–/–^*) (Figure 1A). At 16 weeks of age, *Pkd1^–/–^* mice show overt cystogenesis (14). To determine the presence of complement, we evaluated C3b deposition in kidneys at 16 weeks of age. While barely detectable in WT kidney tubules, C3b deposition was evident in renal tubular epithelial cells from *Pkd1^–/–^* mice (Figure 1, B and C).

Complement cascade activation is tightly controlled by specific soluble and membrane-bound inhibitors, such as DAF and CD59 (8). Compared with WT controls, we found a significant reduction in DAF protein expression in *Pkd1^–/–^* kidneys (Figure 1, D and E), suggesting that spontaneous activation of the alternative complement pathway in ADPKD may be triggered by DAF downregulation. Similarly, the expression of CD59 was reduced in *Pkd1^–/–^* kidneys (Figure 1, F and G) concomitantly with the accumulation of MAC in tubular membranes (Figure 1, H and I). Overall, these data suggest that downregulation of complement regulators DAF and CD59 in *Pkd1^–/–^* kidneys leads to the activation of the complement cascade and to MAC deposition.

### Increased expression of complement genes and downregulation of complement regulators in Pkd1^–/–^ kidneys.

**Figure 2:**
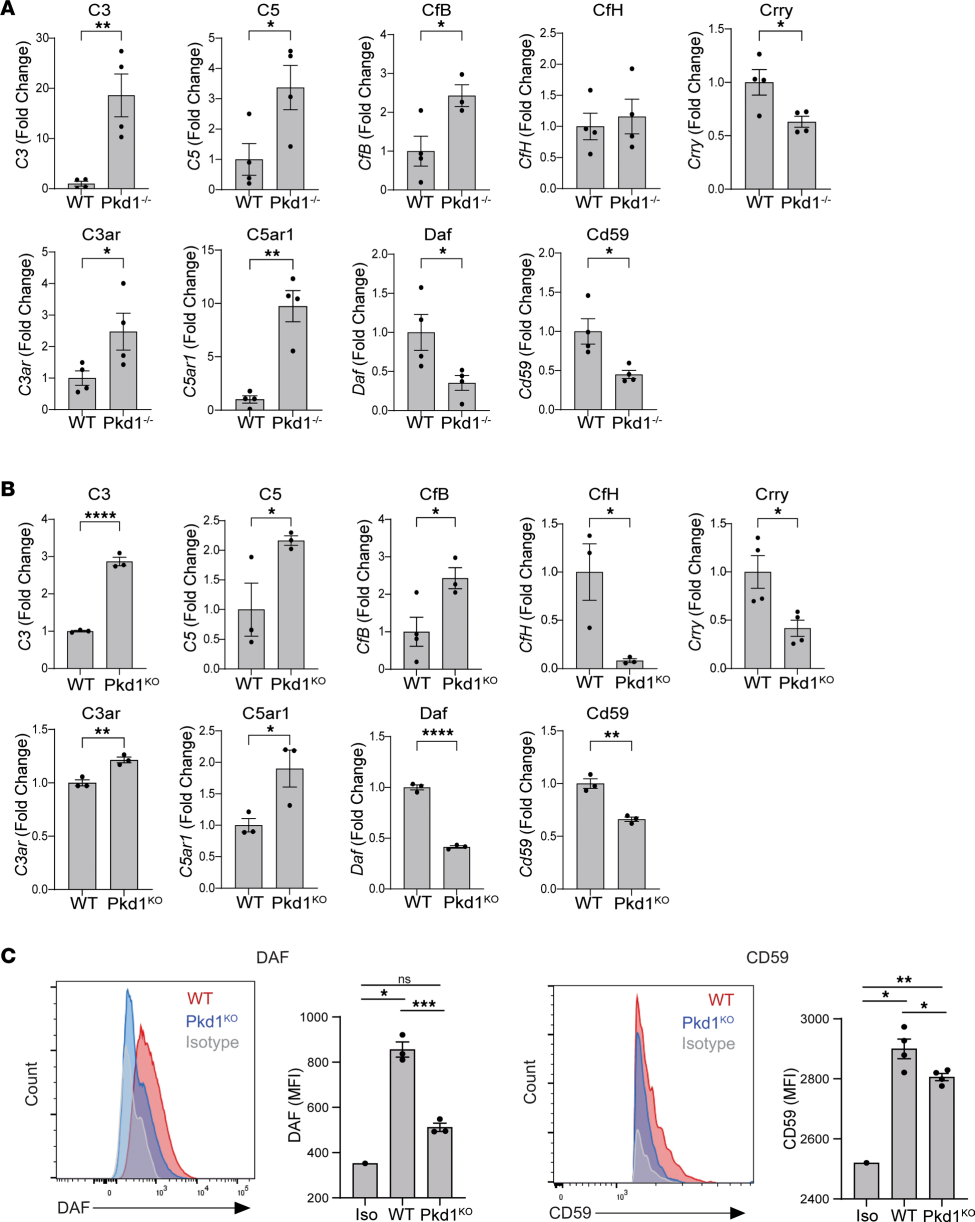
*Pkd1^–/–^* tubular cells are a source of complement components. Complement-related gene expression in (**A**) WT (*n* = 4) and *Pkd1^–/–^* (*n* = 4) kidneys and in (**B**) WT and Pkd1^KO^ tubular cell lines. Each dot represents (**A**) 1 animal or (**B**) the average of separate experiments. (**C**) Representative plots (left) and data quantification (right) of DAF and CD59 expression in WT and Pkd1^KO^ epithelial cells. *P < 0.05; **P < 0.01; ***P < 0.001; ****P < 0.0001 by unpaired, 2-tailed *t* test (**A** and **B**) or 2-way ANOVA with Tukey’s multiple-comparison test (**C**). NS, not significant.

The main source of circulating complement components is the liver, but other tissues, including the kidney, can produce complement proteins (8). Therefore, to determine whether *Pkd1^–/–^* kidneys represent a source of complement, we assessed the expression of complement genes by quantitative real-time PCR (RT-PCR). The results showed that while the genes for all tested complement effector components were expressed in WT kidneys, *C3*, *C5*, and complement factor B (*CfB*) mRNA expression was significantly higher in *Pkd1^–/–^* kidneys (Figure 2A). The expression of complement receptors *C3ar* and *C5ar1* was also significantly increased in *Pkd1^–/–^* kidneys (Figure 2A). While no differences were observed in the mRNA levels of *CfH* between *Pkd1^–/–^* and WT kidneys, the expression of *Daf* and *CD59* genes was significantly reduced in *Pkd1^–/–^* kidneys (Figure 2A), indicating that the parallel reduction in these factors observed on kidney sections (Figure 1, D–G) was at least in part due to transcriptional downregulation. mRNA expression of *Crry*, another complement regulator, was also reduced in *Pkd1^–/–^* kidneys (Figure 2A).

### Pkd1^KO^ tubular cells recapitulate the complement-related gene expression observed in Pkd1^–/–^ kidneys.

To determine the relationship between PC1 expression and complement regulation in renal tubular cells, we used a WT renal collecting duct cell line and the derived Pkd1^KO^ cells, which reproduce the deletion of the *Pkd1^–/–^* model (see ref. 15 for further details).

We observed that complement gene expression in Pkd1^KO^ cells largely recapitulated the data generated in *Pkd1^–/–^* kidneys (Figure 2B), suggesting that the effect on complement genes expression directly results from the loss of *Pkd1* in epithelial cells. Compared with controls, Pkd1^KO^ cells also showed a significant downregulation of *CfH* (Figure 2B), but this difference was not present between *Pkd1^–/–^* and WT kidneys (Figure 2A). We confirmed that the downregulation of *Daf* and *CD59* mRNAs in Pkd1^KO^ cells corresponded to a significant decrease in these complement regulator factors at the protein level (Figure 2C).

Overall, these data suggest that the increased production of complement components and reduced expression of complement regulators could be responsible for the local complement activation in *Pkd1^–/–^* kidneys.

### C3 gene deletion delays renal cystogenesis.

**Figure 3:**
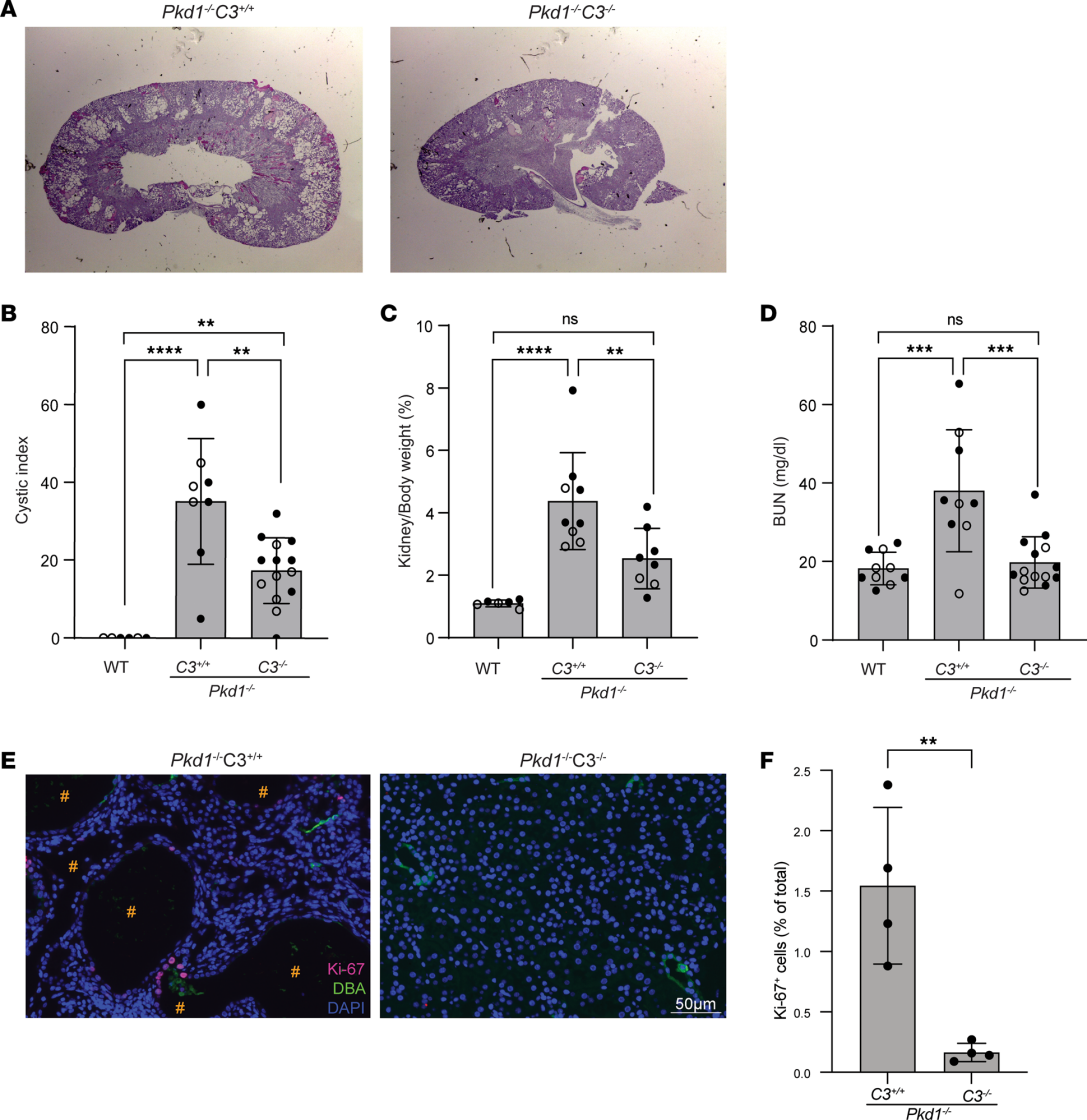
Deletion of *C3* reduces the severity of cystic *Pkd1^–/–^* phenotype. (**A**) Representative PAS staining of kidneys from *Pkd1^–/–^* *C3^+/+^* (*n* = 9) and *Pkd1^–/–^* *C3^–/–^* (*n* = 14) mice. (**B**) Cystic index, (**C**) kidneys/body weight ratio, and (**D**) BUN in WT (*n* = 10), *Pkd1^–/–^* *C3^+/+^*, and *Pkd1^–/–^* *C3^–/–^* mice. Female and male mice are indicated by empty and full circles, respectively. **P < 0.01; ***P < 0.001, ****P < 0.0001 by 1-way ANOVA with Tukey’s multiple-comparison test. NS, not significant. (**E**) Immunofluorescence detection and (**F**) quantification of Ki-67^+^ (red) and DBA^+^ (green) cells in the same mice as in **A**; # indicates cysts. **P < 0.01 by unpaired, 2-tailed *t* test. Scale bars: 50 μm.

To test the causative role of complement activation in ADPKD progression, we crossed *Pkd1^–/–^* mice with *C3^–/–^* mice (both strains are on a B6 background) to generate *Pkd1^–/–^* *C3^–/–^* animals. In these mice, lack of complement component C3 prevents the formation of C3 convertase and downstream generation of C3a, C5a, and MAC. Under normal conditions, *C3^–/–^* mice do not present any specific phenotype, but exhibit increased mortality after infection by group B streptococci (16). We fed *Pkd1^–/–^* *C3^–/–^* mice and *Pkd1^–/–^* *C3^+/+^* controls Dox-supplemented food following the experimental design in Figure 1A. At 16 weeks of age, *Pkd1^–/–^* *C3^+/+^* kidneys appeared overtly cystic, while cystogenesis in *Pkd1^–/–^* *C3^–/–^* kidneys was significantly reduced (Figure 3A). Consistent with the decrease in cyst formation, *Pkd1^–/–^* *C3^–/–^*mice showed lower kidneys/body weight ratios and normal blood urea nitrogen (BUN) values (Figure 3, B–D). No significant sex-specific differences were observed. The significantly lower number of Ki-67^+^ cells in *Pkd1^–/–^* *C3^–/–^* compared with control *Pkd1^–/–^* *C3^+/+^* kidneys indicates reduced cell proliferation in the double knockout (Figure 3, E and F).

Overall, these data demonstrate that the co-deletion of *C3* in *Pkd1^–/–^* mice reduces cystogenesis and preserves renal function.

### C3 deletion associates with reduced inflammation and immune infiltrates in Pkd1^–/–^ kidneys.

**Figure 4:**
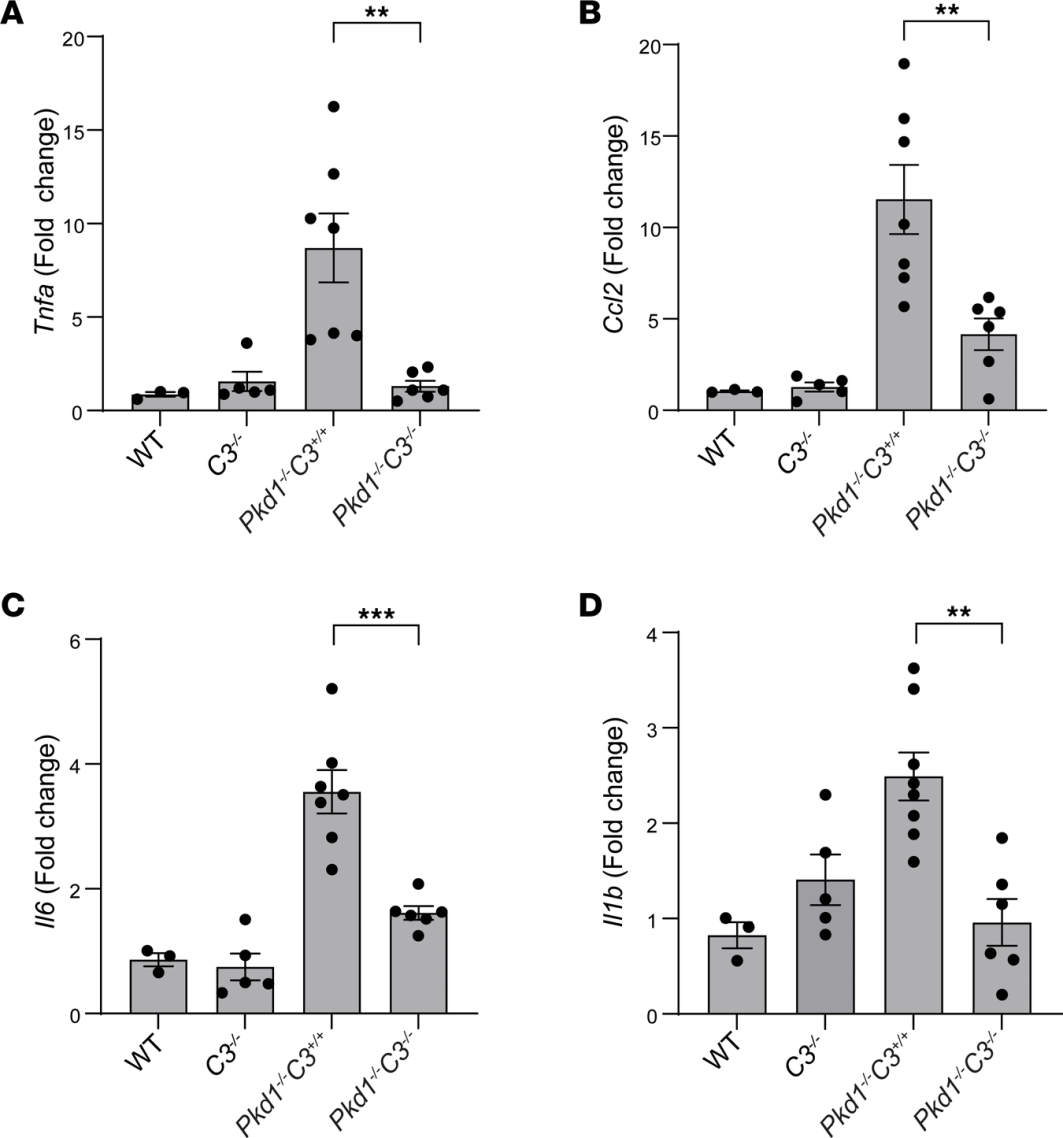
*C3* deletion reduces inflammatory cytokine expression in *Pkd1^–/–^* kidneys. Quantitative RT-PCR for the detection of (**A**) *Tnfa*, (**B**) *Ccl2,* (**C**) *Il6*, and (**D**) *Il1b* gene expression in kidneys from WT (*n* = 3), *C3^–/–^* (*n* = 5), *Pkd1^–/–^* *C3^+/+^* (*n* = 7), and *Pkd1^–/–^* *C3^–/–^* (*n* = 6) mice. Expression is reported as fold change relative to WT control average. **P < 0.01; ***P < 0.001 by 1-way ANOVA with Tukey’s multiple-comparison test.

ADPKD is associated with progressive intrarenal inflammation, a process that is thought to promote cystogenesis (17, 18). When we analyzed the levels of proinflammatory cytokines in whole kidneys, we found that *Pkd1^–/–^* *C3^–/–^* mice expressed significantly lower levels of *Tnfa*, *Ccl2*, *Il6*, and *Il1b* mRNAs than *Pkd1^–/–^* *C3^+/+^* controls (Figure 4, A–D).

**Figure 5:**
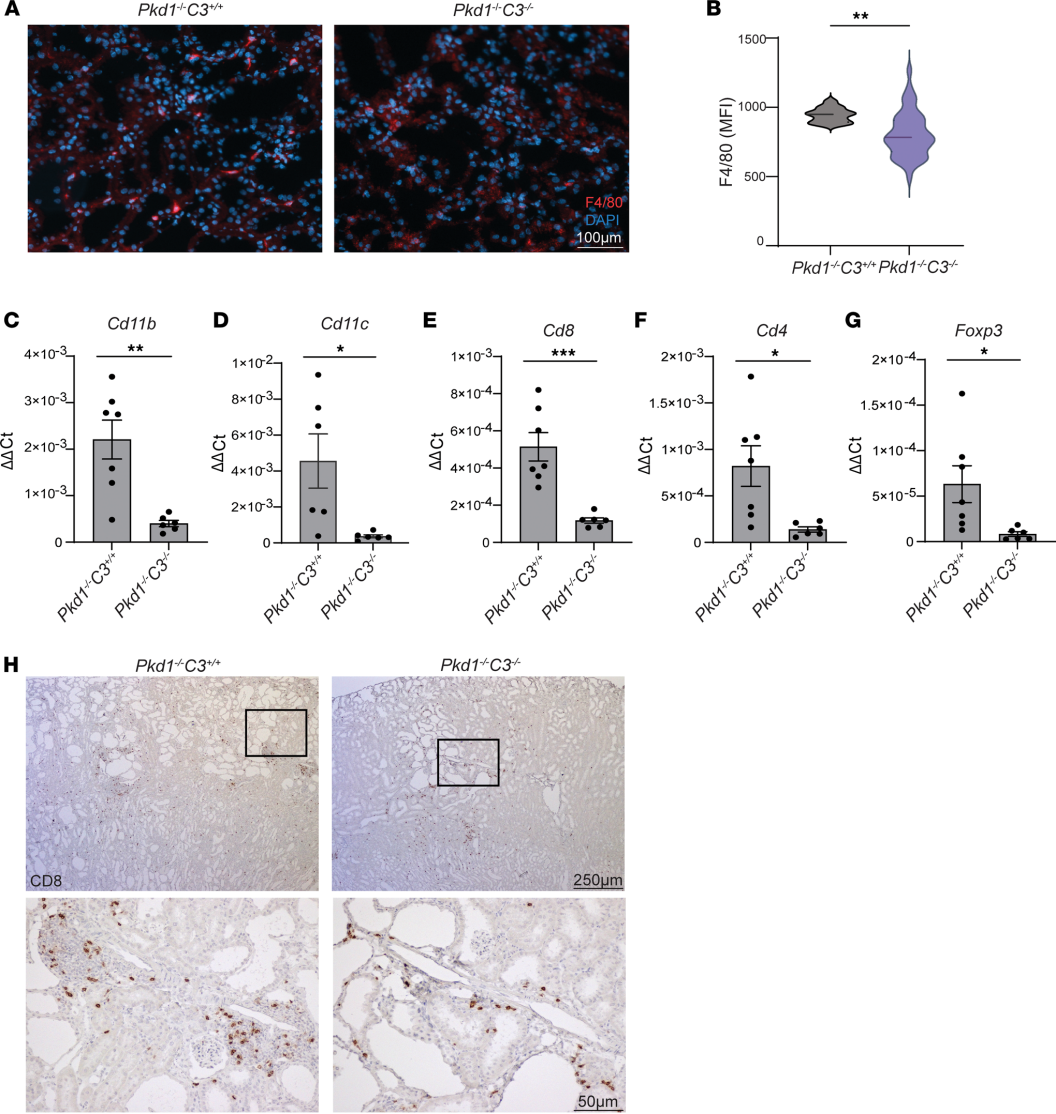
*C3* deletion reduces immune infiltrates in *Pkd1^–/–^* kidneys. (**A**) Immunofluorescent staining and (**B**) relative quantification of F4/80 in kidneys from *Pkd1^–/–^* *C3^+/+^* (*n* = 7) and *Pkd1^–/–^* *C3^–/–^* (*n* = 6) mice. Quantitative RT-PCR assessment of the expression of immune cell gene markers (**C**) *Cd11b*, (**D**) *Cd11c*, (**E**) *Cd8*, (**F**) *Cd4*, and (**G**) *Foxp3* in *Pkd1^–/–^* *C3^+/+^* (*n* = 7) and *Pkd1^–/–^* *C3^–/–^* (*n* = 6) kidneys. Expression is reported as fold change relative to WT control average. (**H**) Representative pictures of T cell infiltrates (CD8^+^) in the same kidneys, with magnified area of the inserts (bottom). *P < 0.05; **P < 0.01; ***P < 0.001 by unpaired, 2-tailed *t* test. Scale bars: 100 μm (**A**), 250 μm (**H**, top), and 50 μm (**H**, bottom).

In agreement with reduced inflammatory cytokine expression, we found that *Pkd1^–/–^* *C3^–/–^* kidneys have significantly lower F4/80^+^ macrophage infiltrates than *Pkd1^–/–^* *C3^+/+^* controls (Figure 5, A and B). Quantitative RT-PCR analyses of *Pkd1^–/–^* *C3^–/–^* kidneys showed lower *Cd11b* mRNA expression, supporting a reduction in macrophage recruitment/expansion in the kidney in the absence of complement activation (Figure 5C), and a decrease in the dendritic cell marker *Cd11c* mRNA (Figure 5D), indicating that complement may also affect other myeloid cell expansion.

Immune infiltrate analyses also showed a reduction in transcripts for CD4^+^ and CD8^+^ T cells and Foxp3^+^ regulatory T cells (Figure 5, E–G). Immunohistochemical staining confirmed lower infiltrates for CD8^+^ T cells (Figure 5H), suggesting that in the absence of C3 the recruitment of immune infiltrates in the kidney is overall decreased.

### C5aR1 activation mediates Pkd1^KO^ cell proliferation.

**Figure 6:**
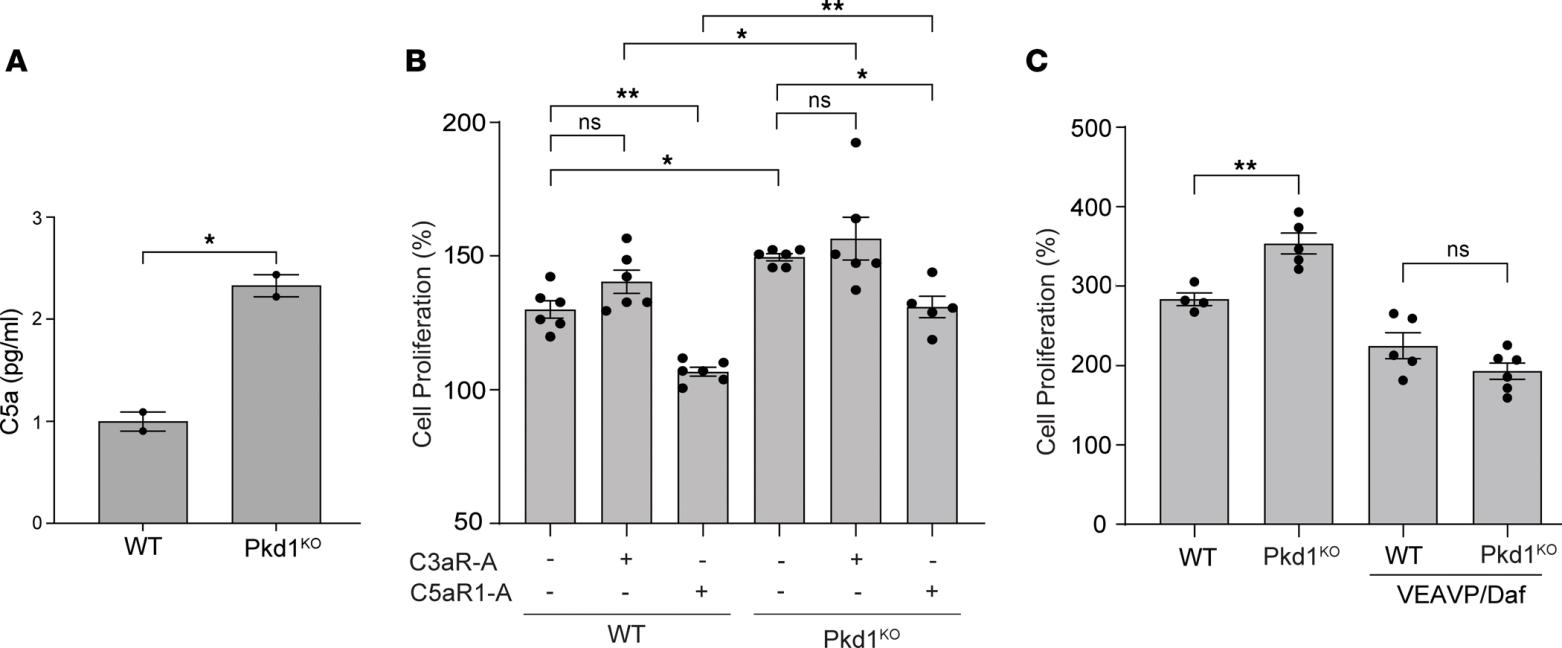
C5aR1 antagonist and DAF overexpression reduce Pkd1^KO^ cell proliferation. (**A**) Levels of C5a (pg/mL) in supernatant obtained from cultured WT and Pkd1^KO^ tubular cells. (**B**) WT and Pkd1^KO^ tubular cell proliferation in the presence or absence of C3aR (10 μM) or C5aR1 (10 μM) antagonist. (**C**) WT, Pkd1^KO^, and *Daf*-transgenic WT/VEAVP-Daf and Pkd1^KO^/VEAVP-Daf tubular cell proliferation. Cell proliferation after 72 hours of culture is represented as percentage of change in cell number relative to number of cells at time 0. *P < 0.05; **P < 0.01 by unpaired, 2-tailed *t* test (**A**) or 2-way ANOVA with Tukey’s multiple-comparison test (**B** and **C**). NS, not significant.

Epithelial cell hyperproliferation supports the expansion of renal cysts and is a hallmark of ADPKD. Pkd1^KO^ cells released higher levels of C5a (Figure 6A), which has been shown to stimulate the growth of other cells, including leukocytes (8). Therefore, we hypothesized that local complement activation in *Pkd1^–/–^* kidneys drives tubular cell proliferation and cyst growth. To test this hypothesis, we measured cell growth following treatment of WT and Pkd1^KO^ cells with C3aR or C5aR1 antagonists. While C3aR antagonism had no significant effect on Pkd1^KO^ cell proliferation,the C5aR1 antagonist significantly rescued the proliferative phenotype of the Pkd1^KO^ cells, confirming the role of local complement activation in supporting the growth of *Pkd1*-deficient cells (Figure 6B).

### DAF overexpression rescues abnormal Pkd1^KO^ cell proliferation.

Because DAF downregulation is a mechanism of local complement activation through the alternative pathway (8), we examined whether increased complement activation and C5a/C5aR1–induced tubular cell proliferation were driven by DAF downregulation in *Pkd1*-deficient cells. We therefore measured the proliferation of Pkd1^KO^ cells following overexpression of DAF.

WT and Pkd1^KO^ cells were transduced with the VEAVP-Daf or VIN16-Daf (Supplemental Figure 1; supplemental material available online with this article; https://doi.org/10.1172/jci.insight.175220DS1) lentivector that allows the constitutive or inducible expression of DAF, respectively. Proliferation of Pkd1^KO^/VEAVP-Daf cells was reduced to the control WT/VEAVP-Daf levels, suggesting that DAF overexpression inhibits the hyperproliferation of Pkd1^KO^ cells by preventing activation of locally produced complement (Figure 6C).

### CTT overexpression rescues DAF expression and abnormal Pkd1^KO^ cell proliferation.

**Figure 7:**
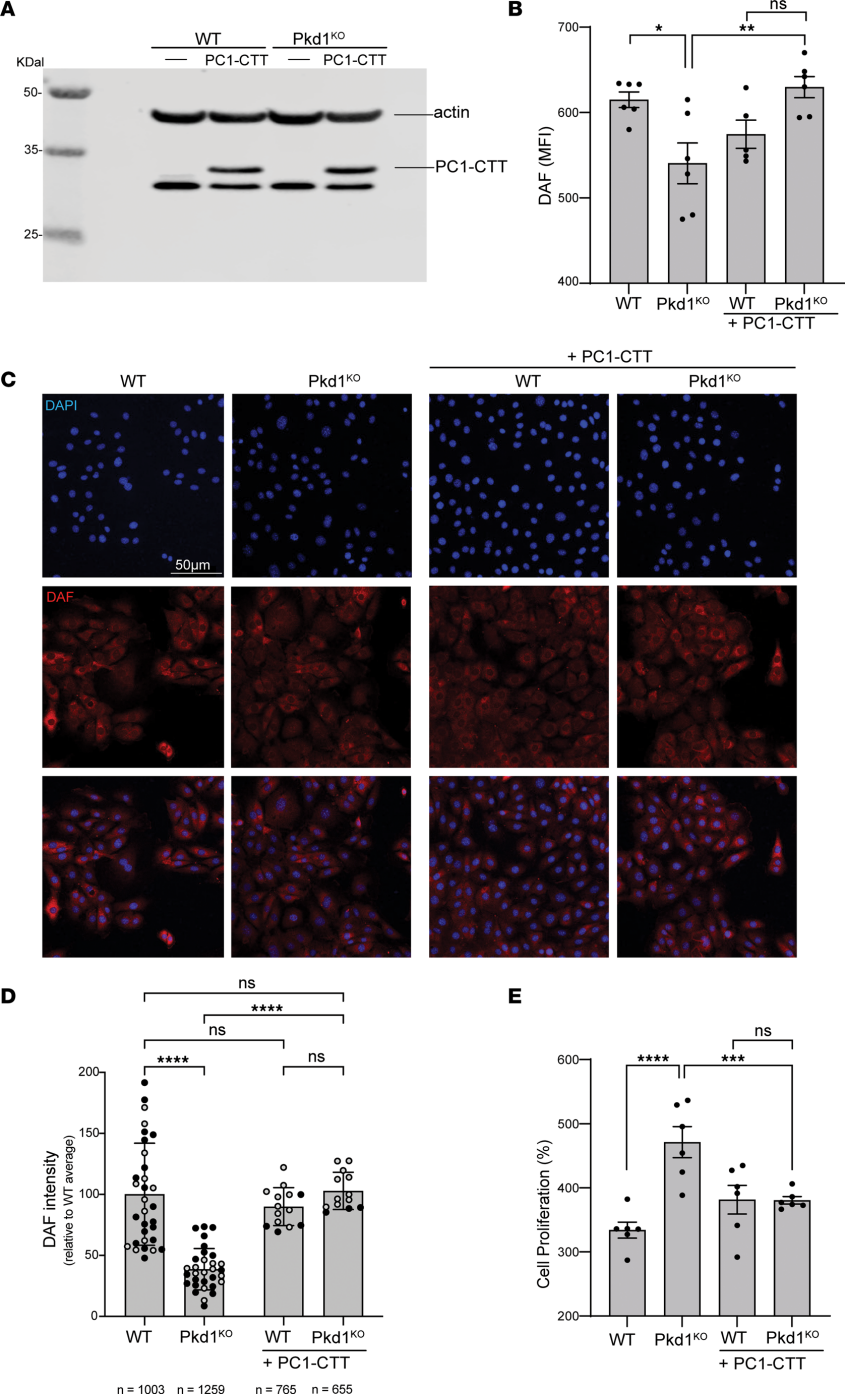
PC1-CTT overexpression rescues DAF expression and abnormal Pkd1^KO^ cell proliferation. (**A**) Western blot of PC1-CTT-S (using anti–S-tag antibody) in lentivirally transduced WT/PC1-CTT and Pkd1^KO^/PC1-CTT cells. Actin expression was measured as loading control. DAF protein expression measured by (**B**) flow cytometry and (**C**) immunofluorescence (original magnification, ×200) was reconstituted in Pkd1^KO^ cells after the exogenous expression of PC1-CTT. (**D**) Quantification of DAF signal by immunofluorescence from 2 independent experiments (gray and black symbols): each data point represents the mean fluorescence per cell from 1 field; *n* is the total number of cells analyzed in each group. (**E**) Proliferation of WT and Pkd1^KO^ cells with or without PC1-CTT. Cell proliferation is represented as percentage change in cell number after 72 hours of culture relative to the number of cells at time 0. ANOVA: *P < 0.05; **P < 0.01; ***P < 0.001; ****P < 0.0001 by 1-way ANOVA with Tukey’s multiple-comparison test. NS, not significant.

The proteolytically cleaved cytoplasmic C-terminal tail of the PC1 protein (PC1-CTT) has been shown to interact with different transcription factors and to be involved in cell proliferation (19–22). To directly test the hypothesis that PC1-CTT loss is responsible for DAF downregulation, WT and Pkd1^KO^ cells were transduced with a lentivector constitutively expressing PC1-CTT (Figure 7A). We found that the expression of PC1-CTT in Pkd1^KO^ cells restored the levels of DAF protein expression (Figure 7, B–D), thus establishing a direct link between PC1-CTT and control of *Daf* expression. Importantly, PC1-CTT expression in Pkd1^KO^ cells normalized their proliferation to WT control levels (Figure 7E), in further agreement with a critical role of DAF downregulation and complement activation in Pkd1^KO^ cell proliferation.

## Discussion

Evidence of complement activation has been reported in murine models of ADPKD and in affected patients (5, 23, 24) and is supported by single-cell RNA sequencing data (25), but the role of complement in cyst growth and the mechanisms underlying this process were unclear. Herein, we showed that genetic deletion of *C3* in a murine ADPKD model ameliorates the cystic phenotype, suggesting that complement activation plays an important role in the pathogenesis of ADPKD. Our findings also indicate that *Pkd1* deletion is associated with an upregulation of complement genes and a downregulation of complement regulator genes in renal tubular cells.

The increased expression of complement-related genes (*C3*, *C5*, *CfB*) and C3b deposition in conditional *Pkd1^–/–^* kidneys and in a Pkd1^KO^ tubular epithelial cell line (15) support the concept that epithelial cells have a role in renal complement production. We further showed that *C5ar1* is significantly upregulated in *Pkd1^–/–^* tubular cells, thus suggesting the possibility of a paracrine or autocrine stimulatory mechanism. In cancer cells (26, 27), the activation of complement can promote cell growth, which is characteristically abnormally sustained in cystic cells. Our data indicate that the growth of Pkd1^KO^ cells is normalized by a specific C5aR1 antagonist, indicating C5a/C5aR1 signaling as a main contributor to the overproliferative phenotype of mutant cells, likely enabled by the significantly higher expression of C5aR1 on these cells. Of note, the fact that C5a was found in the supernatants of WT and, to a higher extent, of Pkd1^KO^ cells indicates that these cells possess the machinery to produce and activate complement products.

Critical to this mechanism is our finding of the reduced expression of the membrane-bound complement regulators DAF and CD59, which associates with unleashed activation of the complement cascade. The pathogenic role of complement regulator downregulation is corroborated by the normalization of Pkd1^KO^ cell growth following DAF overexpression, which further emphasizes the potential role of complement signaling in cystic cells. This effect suggests a mechanistic link between PC1 and DAF expression, as the overexpression of PC1-CTT in Pkd1^KO^ cells normalizes DAF expression.

These conclusions are supported by our in vivo data showing that genetic deletion of *C3* significantly slows cyst development in *Pkd1^–/–^* mice. C3 is a critical component of the complement cascade, and its deletion prevents formation of C3a, C5a, and deposition of MAC on cell membranes. Our data confirm and extend prior evidence that rosmarinic acid, a nonspecific inhibitor of the complement system, improves disease severity in murine ADPKD models (24). Therefore, these findings indicate that the deposition of complement split products in ADPKD kidneys and the presence of complement components in cystic fluid (5, 23, 24) are not a mere downstream effect of progressive kidney tissue destruction and inflammation, but a key element fueling cyst growth.

Inflammatory molecules and macrophage infiltrates have been detected in kidneys of individuals with ADPKD and in animal models of the disease (18, 28, 29) and are thought to play a role in cystogenesis. We found that reduced cystogenesis observed in *Pkd1^–/–^* *C3^–/–^* mice was associated with reduced inflammatory cytokine expression and macrophage infiltrates. Therefore, it is possible that the activation of the C3 complement cascade may be part of a response to injury involving inflammatory signals and immune cell activation that is required for the proper repair mechanisms and reconstitution of the tubular architecture. The uncontrolled activation of complement in the absence of PC1 may disrupt this homeostatic role and favor the progression of cystogenesis.

Our observations support what we believe is a novel interpretation of the regulation of complement in ADPKD, showing that loss of *Pkd1* directly affects DAF expression, complement production, and local activation. While the role of systemic versus local regulation of complement and the contribution of specific cell types to the cystogenic process remain to be dissected, our results indicate that C5a accumulation and signaling through C5aR1 on tubular cells is a key mechanism of proliferation of Pkd1^KO^ cells.

Overall, our findings unravel a causal relationship between complement activation and cystogenesis. In light of the growing armamentarium of complement-targeting molecules, including C5aR1 antagonists with excellent safety profile, we anticipate that our studies will provide important mechanistic information and preclinical data for clinical testing the hypothesis that inhibition of complement activation or signaling retards the progression of ADPKD.

## Methods

### Sex as a biological variable.

Our study examined male and female animals, and similar findings are reported for both sexes.

### Mice and procedures.

WT C57BL/6J (B6), *Pkd1^fl/fl^* (B6.129S4-*Pkd1^tm2Ggg^*/J), and *C3^–/–^* mice were purchased from The Jackson Laboratory. Male and female *Pax8^rtTA^;TetO-Cre;Pkd1^fl/fl^* mice were obtained from the Baltimore Core Center at the University of Maryland and maintained on the C57BL/6J background. To ablate the floxed *Pkd1* alleles, 4-week-old mice were fed Dox-supplemented chow for 4 weeks. Mice were then sacrificed at 16 weeks of age (12 weeks after the first Dox administration). PCR genotyping was performed using the primers indicated in Supplemental Table 1. Investigators involved in quantifying mouse outcomes were blinded to mouse genotype.

### Tissue preparation and histology.

At sacrifice, mice were anesthetized with a 100 μL intraperitoneal injection of sterile ketamine (16 mg/mL, Vedco) and xylazine (7 mg/mL, Akorn, Inc) in PBS (Thermo Fisher Scientific), and kidneys were immediately harvested and frozen in optimal cutting temperature compound (Tissue-Tek O.C.T., Sakura Finetek) or perfused with 4% paraformaldehyde (Thermo Fisher Scientific) in PBS via intracardiac injection at a rate of 8–10 mL/min and paraffin embedded. Histological analysis was performed on dewaxed paraffin sections of kidneys (4 μm thick) that were hydrated and stained with periodic acid–Schiff (PAS) reagent.

### Immunohistochemistry.

Deparaffinized tissue sections were subjected to high-temperature antigen retrieval with Trilogy (Cell Marque) in a pressure cooker. Endogenous peroxidase activity was quenched with 0.03% H_2_O_2_ for 10 minutes, and nonspecific protein interactions were inhibited by incubation with serum-free protein block (Dako). The slides were then incubated with a rabbit monoclonal antibody against CD8 (ab209775, Abcam) at a 1:1000 dilution. Goat anti-rodent and rabbit anti-rodent HRP-Polymers (GHP516, RMR622, Biocare Medical) followed by DAB chromogen were used to develop the staining.

### Immunofluorescence.

Tissue-Tek O.C.T.–preserved cryosections (5 μm thick) were fixed with 4% periodate-lysin-paraformaldehyde fixative in PBS for 15 minutes and then washed with PBS 3 times for 5 minutes and left for 60 minutes at room temperature with blocking solution containing PBS, 2% BSA (Thermo Fisher Scientific), 2% FBS (Thermo Fisher Scientific), and 0.2% fish gelatin (Sigma-Aldrich). Immunofluorescent staining was performed using the following primary antibodies: anti-DAF (rabbit anti-mouse, 1:50; MA5-29678, Thermo Fisher Scientific), anti-C3b (rat anti-mouse, 1:50; HM1065, Hycult Biotech), anti–C5b-9 (rabbit anti-mouse, 1:100; bs-2673R, Bioss), anti-CD59 (rabbit anti-mouse, 1:100; MA5-32588, Thermo Fisher Scientific), and anti-F4/80 (rabbit anti-mouse, 1:20; 28443-1-AP, Proteintech) at 4°C overnight. Sections were then washed and incubated with appropriate secondary antibody for 60 minutes at room temperature: goat anti-rabbit Alexa Fluor 488 (1:200, A11008, Thermo Fisher Scientific), goat anti-rabbit Alexa Fluor 568 (1:200, A11011, Thermo Fisher Scientific), and goat anti-rat Alexa Fluor 488 (1:200, A11006, Thermo Fisher Scientific). Nuclei were finally counterstained with DAPI mounting media (ProLong Gold antifade reagent with DAPI; P-36931, Thermo Fisher Scientific). To stain for Ki-67, after deparaffinization and rehydration of the tissue, antigen retrieval was performed using pH 9.0 Tris-EDTA buffer. Sections were treated with 3% H_2_O_2_ for 10 minutes at room temperature to block endogenous peroxidase activity and then incubated in 5% normal goat serum for 30 minutes in PBS at room temperature. Sections were incubated with rabbit anti–mouse Ki-67 (1:200; ab15580, Abcam) overnight at 4°C, followed by incubation with Cy3-conjugated donkey anti-rabbit IgG (1:200; 711-165-152 Jackson Immunoresearch) and fluorescein-labeled *Dolichos biflorus* agglutinin (DBA) (1:200; FL-1031, Vector Laboratories) for 2 hours at room temperature. Sections were then incubated in 0.1% Sudan black B in 70% ethanol to reduce background fluorescence and finally mounted in DAPI fluoromount-G (0100-20, SouthernBiotech). Antibody binding was estimated by constructing a contour mask on the bright-field image. Whole sections for each kidney were acquired using a fluorescence non-confocal laser scanning microscope (Zeiss AxioImager Z2M with ApoTome.2). Zen software was used to quantify DAF, C3b, CD59, C5b-9, and F4/80 staining intensity. The mean fluorescence intensity (MFI) was quantified for several areas for each section. Because the DAF, CD59, and C5b-9 signals appeared diffuse, the MFI was normalized to that of DAPI.

For immunofluorescence, epithelial cells were fixed with 4% paraformaldehyde for 15 minutes, permeabilized with 0.5% Triton X-100 in PBS for 10 minutes at room temperature, washed in PBS, and then blocked with 3% BSA and 1% normal goat serum in PBS for 60 minutes at room temperature. Incubation with rabbit anti–mouse DAF primary antibody (MA5-29678, Thermo Fisher Scientific) was carried out for 2 hours at room temperature. Following 3 PBS washings, incubation with the Cy3-conjugated goat anti-rabbit secondary antibody (111-165-144, Jackson Immunoresearch) was performed for 1 hour at room temperature. Cells were finally washed with PBS and mounted with DAPI Fluoromount-G. DAF signal intensity per cell was measured using Fiji (https://imagej.net/).

### Cystic index, kidneys/body weight ratio, and BUN measurement.

The cystic index was determined by assessing the cyst area relative to the total sample’s surface using ImageJ software (NIH). Two kidneys per body weight were used to determined the ratio at the time of sacrifice. BUN was measured from sera collected by submandibular vein or cardiac puncture at sacrifice, using the QuantiChrom Urea Assay Kit (DIUR-100, Bioassay Systems) according to the manufacturer’s instructions.

### Cell culture.

WT collecting duct epithelial cells were immortalized from *Pkd1^fl/fl^* (B6.129S4-*Pkd1^tm2Ggg^*/J) kidneys and Pkd1^KO^ cells were derived from WT upon transduction with a Cre-expressing lentiviral vector, as we previously described (15). All cells were maintained in DMEM/F12 (R&D Systems) supplemented with 5% FBS and 2 mM L-glutamine (R&D Systems) and grown at 37°C in 5% CO_2_ atmosphere.

### Lentivector generation.

Replication-incompetent lentivectors were produced via polyethylenimine-mediated (Polysciences, Inc.) cotransfection of 293T cells with viral constructs and psPAX2 and pMD.G2 packaging plasmids (Addgene). Virus-containing supernatants were concentrated by centrifugation and transduction was carried out in the presence of 10 μg/mL polybrene. Antibiotic selection was started 24 hours after transduction to establish stable polyclonal populations.

The mouse *Daf* coding sequence was inserted into the puromycin-selectable VEAVP lentivector under the control of the constitutive *EF1* promoter. Following transduction of WT and Pkd1^KO^ cells with VEAVP-Daf and puromycin selection, the DAF-overexpressing polyclonal cell lines WT/VEAVP-Daf and Pkd1^KO^/VEAVP-Daf were derived.

The fragment coding for the last 200 amino acids of PC1 (PC1-CTT) amplified from the full-length mouse *Pkd1* cDNA (30) and S-tagged at the 3′ end was cloned in the hygromycin-selectable VEAVY lentiviral vector to generate VEAVY/PC1-CTT-S. This was used to transduce WT and Pkd1^KO^ cells, and upon hygromycin selection the polyclonal populations WT/PC1-CTT and Pkd1^KO^/PC1-CTT were obtained in which PC1-CTT-S is constitutively expressed.

### Cell proliferation assay.

In selected experiments, cells were treated with C3aR antagonist SB 290157 or C5aR1 antagonist PMX53 (10 μM, MilliporeSigma) at the time of seeding. Untransduced or transduced WT and Pkd1^KO^ cells were cultured for 72 hours in the presence or absence of C3aR antagonist SB 290157 or C5aR1 antagonist PMX53 (10 μM) and then washed with 1× PBS and fixed in 4% paraformaldehyde in PBS for 15 minutes. After washing with distilled water, staining with 0.1% crystal violet (Sigma-Aldrich) was carried out for 20 minutes, followed by 3 more washes with distilled water. An equal volume of 10% acetic acid was added to each well and cells were incubated at room temperature for 20 minutes while shaking. Samples were diluted with water (1:4) to measure absorbance at 590 nm. Proliferation was determined as the increase in cell number after 72 hours of culture relative to the number of cells at time 0.

### Quantitative RT-PCR.

Total RNA was isolated from whole mouse kidneys or tubular cell lines using TRIzol (Thermo Fisher Scientific) or a Directzol RNA miniprep kit (Zymo). cDNA was synthesized using cDNA EcoDry Premix (Clontech). Quantitative RT-PCR assays were performed using the TaqMan Universal PCR Master Mix and primer sets shown in Supplemental Table 2.

Quantitative RT-PCRs for *Tnfa*, *Ccl2*, *Il6*, and *Il1b* were performed with SYBR Green (Applied Biosystem). Relative expression was normalized to that of the housekeeping *Gapdh* or *Rps11* gene for analysis (Supplemental Table 2).

### Flow cytometry.

We used standard approaches for surface and intracellular staining as previously published (31). For surface and intracellular staining, we used PE-conjugated hamster anti–mouse CD55 (clone RIKO-5 [RUO], BD Pharmigen) and FITC anti–mouse CD59 monoclonal antibody (clone 1F5, Thermo Fisher Scientific), on whole cells or cells permeabilized using an eBioscience Foxp3/Transcription Factor Staining Buffer Set (Thermo Fisher Scientific), respectively. Data were acquired (at least 10,000 to 100,000 events, in most cases >100,000 events) on a 3-laser Canto II flow cytometer (BD Biosciences) and analyzed using FlowJo software (https://www.flowjo.com).

### Western blots.

Cells were lysed in RIPA buffer (10 mM Tris-Cl pH 8.0, 140 mM NaCl, 1 mM EDTA, 0.5 mM EGTA, 1% Triton X-100, 0.1% sodium deoxycholate, 0.1% SDS) supplemented with phosphatase-protease inhibitors (A32961, Thermo Fisher Scientific). Lysates were run in 4%–12% Bis-Tris gels in MES-SDS running buffer and transferred to PVDF membranes, which were then blocked using 5% skim milk in Tris-buffered saline (TBS) containing 0.1% Tween 20 (TBS-T), at room temperature for 1 hour. Incubation with primary antibodies mouse anti–S-tag (MA1-981, Invitrogen) and mouse anti–β-actin–HRP (sc-47778, Santa Cruz Biotechnology) was carried out overnight at 4°C in TBS-T with 5% BSA followed by TBS-T washes and incubation with the appropriate HRP-conjugated secondary antibodies for 1 hour at room temperature. After final washes in TBS-T, membranes were incubated with Supersignal West Femto (34096, Thermo Fisher Scientific) and light signals were detected using an imaging system (LI-COR Biotechnology).

### C5a quantification.

Cells were seeded at 2 × 10^6^ per plate and after culture in complete medium for 24 hours they were washed with PBS twice and switched to serum-free medium for another 24 hours. Twenty milliliters of conditioned supernatants from 2 independent experiments were concentrated 65 times using Amicon Ultra centrifugal filters (UFC900508, Millipore) following the manufacturer’s instructions. C5a levels were quantified in concentrated cell supernatant by ELISA (LS-F537-1, LSBio), according to the manufacturer’s instructions.

### Statistics.

Continuous variables are reported as mean ± SD. We used unpaired, 2-tailed *t* tests for 2-group comparisons or 1-way or 2-way ANOVA (with Tukey’s test for post hoc pairwise differences) for multiple independent group comparisons. *P* values less than 0.05 were regarded as statistically significant. All statistical analyses were performed using Prism (GraphPad Software Inc).

### Study approval.

Animal experiments were performed with the approval of the Institutional Animal Care and Use Committee (IACUC) of Icahn School of Medicine at Mount Sinai in New York City.

### Data availability.

Raw data for all data points shown in graphs are provided in the supplemental Supporting Data Values file. For original data, please contact paolo.cravedi@mssm.edu or luca.gusella@mssm.edu.

## Author contributions

SB and MY share first authorship due to their overall contribution to the paper. SB is listed first due to the leading role in the study. SB, MY, PM, MG, KB, YK, and GLG performed experiments and analyzed data. CC, GLM, WMB, and ND critically revised and edited the manuscript. PC and GLG conceptualized and oversaw the project, acquired funding, wrote and edited the manuscript. All the authors approved the final version of the manuscript.

## Supplementary Material

Supplemental data

Unedited blot and gel images

Supporting data values

## Version 1 06/24/2024

Electronic publication
