# Supplementary material for: Reduced decay-accelerating factor expression promotes complement-mediated cystogenesis in murine ADPKD
Source: JCI Insight. 2024 May 23;9(12):e175220. doi: 10.1172/jci.insight.175220 (PMC11383362; doi:10.1172/jci.insight.175220)
Supplement: Supplemental data [file jciinsight-9-175220-s154.pdf]

**Supplemental Materials**

- **Supplemental Table S1.** Primers used for mouse genotyping: page 2
- **Supplemental Table S2.** Primers used for mouse genotyping: page 2
- **Supplemental Figure 1.** DAF transgenic expression.

- **Supplementary Table S1. Primers used for mouse genotyping.**

| **Genotyping Primers** | | |
|---|---|---|
| **Gene** | **Forward Primer** | **Reverse Primer** |
| *C3-1 WT* | AGGGCGAGAACTGCATAAAGG | GTTGTTCACGCAGCTTGGT |
| *C3* KO | CCAGGGCGAGAACTGCAT | CAGACTGCCTTGGGAAAAGC |
| *Pax8* | CCATGTCTAGACTGGACAAGA | CTCCAGGCCACATATGATTAG |
| *Pkd1* | CCTGCCTTGCTCTACTTTCC | AGGGCTTTTCTTGCTGGTCT |
| *Cre* | ATTGCTGTCACTTGGTCGTGGC | GGAAAATGCTTCTGTCCGTTTGC |

**Supplementary Table S2. Primers for Quantitative RT-PCR.**

| **Gene Expression Primers** | | |
|---|---|---|
| **Gene** | **Assay ID** | **Brand** |
| *C3* | Mm01232779_m1 | Thermo Fisher Scientific |
| *C5* | Mm00439275_m1 | Thermo Fisher Scientific |
| *CfB* | Mm00433909_m1 | Thermo Fisher Scientific |
| *CfH* | Mm01299248_m1 | Thermo Fisher Scientific |
| *C3aR* | Mm02620006_s1 | Thermo Fisher Scientific |
| *C5aR1* | Mm00500292_s1 | Thermo Fisher Scientific |
| *Daf* | Mm00438377_m1 | Thermo Fisher Scientific |
| *Cd59* | Mm00483149_m1 | Thermo Fisher Scientific |
| *Crry* | Mm00785297_s1 | Thermo Fisher Scientific |
| *Cd11c* | Mm00498698_m1 | Thermo Fisher Scientific |
| *Cd11b* | Mm00434455_m1 | Thermo Fisher Scientific |
| *Cd8* | Mm01182107_g1 | Thermo Fisher Scientific |
| *Cd4* | Mm00442754_m1 | Thermo Fisher Scientific |
| *Foxp3* | Mm00475162_m1 | Thermo Fisher Scientific |
| *Gapdh* | Mm99999915_g1 | Thermo Fisher Scientific |
| **Gene** | **Forward Primer** | **Reverse Primer** |
| *Ccl2* | GACCCGTAAATCTGAAGCTAA | CACACTGGTCACTCCTACAGAA |
| *Il-1b* | GCAACTGTTCCTGAACTCAACT | ATCTTTTGGGGTCCGTCAACT |
| *Il-6* | GCACCTCAGATTGTTGTTG | AAATAGTGTCCTAACGCTCATAC |
| *Tnfa* | TCACTGGAGCCTCGAATGTC | GTGAGGAAGGCTGTGCATTG |
| *Rps11* | CGTGACGAAGATGAAGATGC | GCACATTGAATCGCACAGTC |

A

B

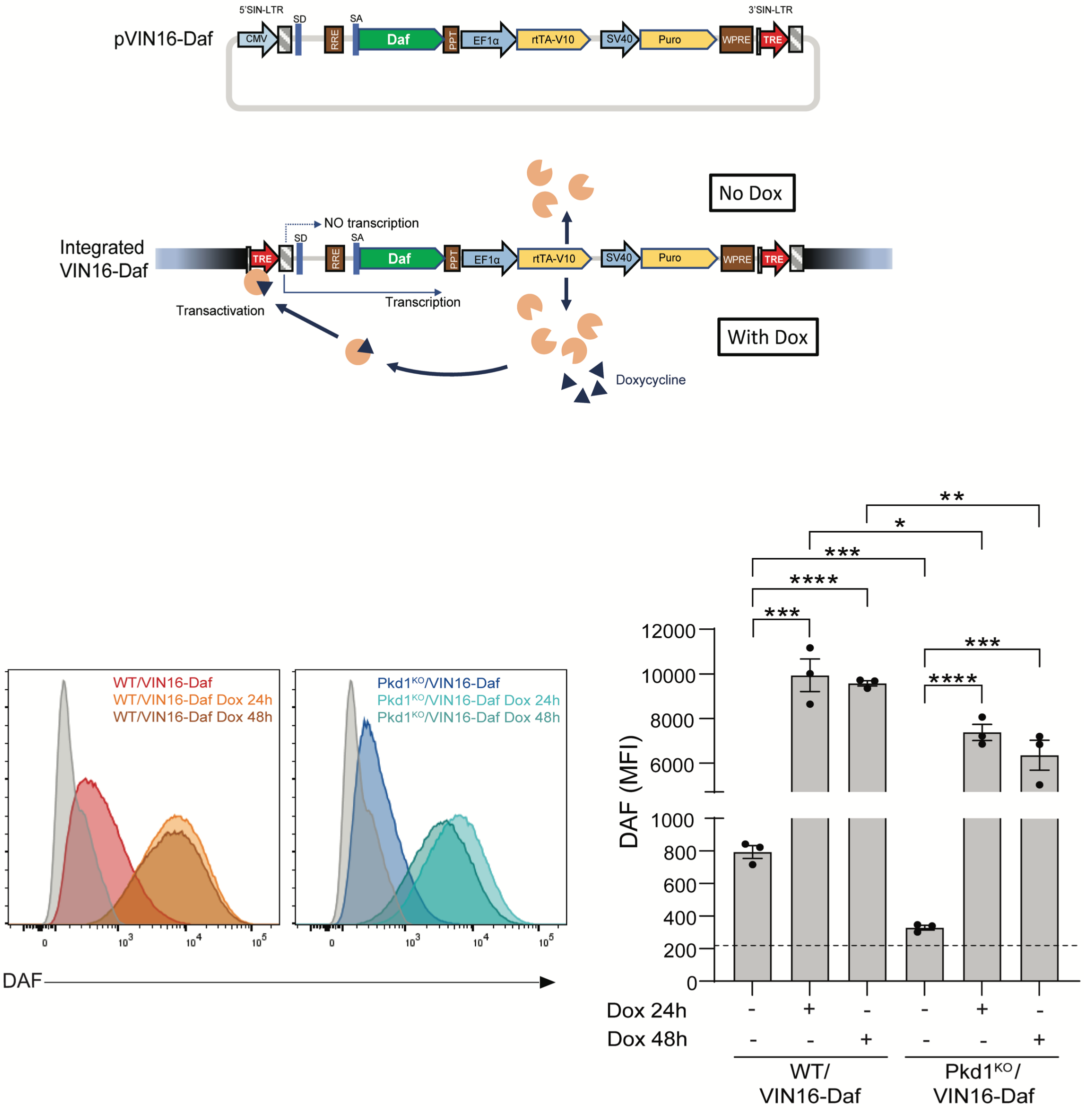


**Supplemental Figure 1. DAF transgenic expression.** (**A**) Representative scheme of puromycin selectable VIN16-Daf lentivector construct. (**B**) Representative plots and (**C**) data quantification of DAF in WT/VIN16-Daf and $PKD^{KO}$/VIN16-Daf tubular cells with or without Doxocycline (Dox). Dotted line: isotype. ANOVA *P < 0.05; ****P < 0.0001.
