# Supplementary material for: Reduced decay-accelerating factor expression promotes complement-mediated cystogenesis in murine ADPKD
Source: JCI Insight. 2024 May 23;9(12):e175220. doi: 10.1172/jci.insight.175220 (PMC11383362; doi:10.1172/jci.insight.175220)
Supplement: Unedited blot and gel images [file jciinsight-9-175220-s155.pdf]

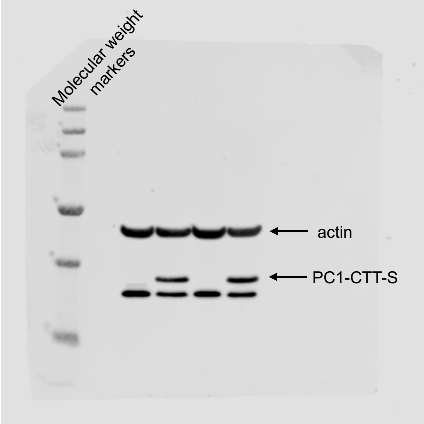

Figure 7A. Full image. Following lysates transfer, PVDF membrane was incubated with anti S-Tag antibody and anti-actin antibodies. The lowest band is a ubiquitously expressed protein recognized by the antibody (likely an endogenous ribonuclease containing the sequence from which the S-tag is derived) that is not affected by the transduction.
